# First study on stress evaluation and reduction in hospitalized cats after neutering surgery

**DOI:** 10.14202/vetworld.2022.2111-2118

**Published:** 2022-09-05

**Authors:** Worranan Klintip, Thitichai Jarudecha, Khwankamon Rattanatumhi, Sudpatchara Ritchoo, Rattana Muikaew, Sakkapop Wangsud, Metita Sussadee

**Affiliations:** Department of Veterinary Technology, Faculty of Veterinary Technology, Kasetsart University, Bangkok, Thailand

**Keywords:** cortisol, domestic cats, food intake, pheromone

## Abstract

**Background and Aim:**

In Thailand, domestic cats are the most common companion animal, and many are admitted to veterinary clinics for neutering surgery; however, such environment can induce stress. This is the first study to evaluate stress in hospitalized cats after neutering surgery using cat stress score (CSS) and salivary cortisol levels, including the impact of providing a hiding box (B) and/or administering a pheromone product to reduce stress.

**Materials and Methods:**

The study design was based on a randomized controlled clinical trial. A total of 80 domestic cats undergoing routine neutering surgery were assessed for their behavioral demeanor scoring system (DSS) as friendly (DSS1) and aggressive (DSS2) based on a DSS. During admission, the cats were randomly allocated to single standard cages with one of the following treatments: (B), feline facial pheromone (P), a combination of hiding box and the pheromone (BP), or no additional enrichment (C). Cat stress score, food intake, and hide-seeking behavior were recorded. The cortisol enzyme-linked immunosorbent assay kit was used to assess the salivary cortisol level.

**Results:**

On the 1^st^ day of admission, aggressive cats had a significantly higher CSS (4.16 ± 0.29) than friendly cats (3.27 ± 0.16). Both demeanor cat groups showed statistically significant reductions in stress levels earlier than the control group after providing the enrichments. Saliva cortisol measurements ranged from 0.24 to 0.66 ng/mL. No statistical differences in cortisol levels were observed between the 1^st^ day and other days of admission. In contrast, no differences in food intake and hide-seeking behavior were seen within each group during the same period.

**Conclusion:**

Results suggested that stress and stress responses in cats depended on behavioral demeanor. The provision of enrichment, including hiding box and feline facial pheromone in singly housed caging reduced stress, especially in aggressive cats. However, salivary cortisol analysis, food intake, and hide-seeking behavior were ineffective for assessing stress in cats after neutering surgery.

## Introduction

Recent statistics from the Department of Livestock Development, Thailand, estimated that out of more than 800,000 registered cats in 2020, 93.56% were owned cats and 6.44% were free roaming [1]. Stray cats are vulnerable to poor welfare, which can have serious health consequences. An important cat owner practice that can help reduce the number of stray cats is sterilization, often known as spaying/neutering. The benefits of neutering male cats include better welfare and an overall decrease in mortality, while neutering female cats prevent pyometra and mammary tumors [2]. A survey conducted by Culture and Human-Animal Interactions in 2015 showed that 19% of cats from 23% of the respondents were sterilized [3]. Various environmental aspects may affect the welfare and stress levels of a cat brought to a veterinarian for sterilization. Owners are often reluctant to take their cats to a veterinarian due to stress from transportation to-and-from the clinic, including increased stress levels while staying at the veterinary clinic [4]. Among the cat owners, 56.7% are unaware of the overall stress levels of their cats [5]. From leaving the house to returning home, all phases of the veterinary visit were found negatively impact on cat welfare; hence, the clinic was deemed more stressful than the home [6].

During cat exposure to novel environments, stress develops, and the central nervous system responds to this stimulus which alters the behavior and affects the autonomic nervous system. The hypothalamic-pituitary-adrenal axis (HPA) and the sympathetic nervous system are activated in response to stress and the adrenocorticotropic hormone and cortisol concentrations increase [7]. Stress is, therefore, an adaptive response essential for favorable feedback to harmful stimuli through metabolic, endocrine, hemodynamic, behavioral, and immunological systems [8]. The persistent exposure to stress impacts increased sensitivity to pain, longer post-surgical recovery, sepsis, or delayed healing [9]. Stress parameters such as heart rate, respiratory rate, and blood pressure significantly increased in healthy cats during transfer between the home and veterinary clinic [10]. To attenuate this stressful event, building the cat’s tolerance to handling and veterinarian examinations are paramount [11]. Various methods for measuring stress in cats include behavioral, physiological, and immunological measurements, including sickness behaviors and viral infections. In several studies, the cat stress score (CSS), hiding observation, food intake, and body weight monitoring are standardized methods for behavioral assessment of stress in cats [12–15]. Cats with high-stress scores likely develop a susceptibility to disease, especially upper respiratory tract infection. Moreover, caging-related stress might result in decreased food intake and weight loss [13]. Most physiological studies of stress in cats measure activation of the HPA axis and changes in circulating concentrations of cortisol [16–18]. Salivary cortisol measurement is less invasive than blood sampling and can potentially reflect short-term stress in the same way as plasma cortisol levels [19]. The cage environment was enriched by providing opportunities to hide, such as a hiding box and/or partially covering the cage front, which can reduce fear and stress in cats [15, 20–23]. Leij *et al*. [24] found that hiding enrichment also minimized behavioral stress in shelter cats. The synthetic pheromone Feliway^®^ was used in this study to make the cats feel safer and reduce stress in an unfamiliar environment [25]. The F3 feline facial pheromone is believed to calm cats in stressful situations or new environments by mimicking territorial markings [26]. Furthermore, the pheromone decreased stress and sneezing in kittens with FHV-1 [27]. However, in shelter cats exposed for 21 days, the product did not reduce stress scores [28]. The response to each environmental enrichment depended on the cat’s behavioral style [23]. A cat demeanor scoring system (DSS) has been used in many previous behavioral research studies to classify cat temperament [29, 30], which veterinarians can also use. The DSS consists of multiple-choice questions describing the cat’s behavior during each interaction. The DSS classified the cats into five different demeanor categories as follows: (1) Friendly and confident, (2) friendly and shy, (3) withdrawn and protective, (4) withdrawn and aggressive, and (5) overtly aggressive. The DSS was used to detect changes in behavior in healthy cats undergoing short-term stays in a veterinary clinic [29] and during the perioperative (closed castration) period [30]. To date, no scientific studies have investigated stress in cats after neutering surgery based on the cat’s demeanor. There are also no published reports that describe the use of a DSS combined with the provision of environmental enrichment to evaluate stress in cats at veterinary clinics. This study classified cats as either friendly or aggressive using the modified DSS. It provided different enrichment treatments that were cost-effective and easy to implement to improve cat welfare, such as a hiding box and pheromone treatment. The CSS and saliva cortisol levels, one of the stress indicator hormones, were used to evaluate stress levels in the control and treatment groups.

This study aims to evaluate stress in hospitalized cats after neutering surgery and the impact of providing a hiding box and/or administering a pheromone product to reduce stress. We hypothesized that enriching the environment of cats at veterinary clinics after neutering surgery using a combination of hiding box and synthetic pheromone treatment would reduce the stress score and lower saliva cortisol concentration.

## Materials and Methods

### Ethical approval

Ethical approval for the study was granted by the Ethics Committee of the Kasetsart University Research and Development Institute (ACKU64-VTN-007). All cat owners were asked to sign consent forms for the observational clinical study.

### Study period and location

The study was conducted from May 2020 to December 2021 in the Faculty of Veterinary Technology, Kasetsart University.

### Study animals

Sample size was calculated using the formula: n = ([z_1-_^α^_/2_ + z_1-_^β^]^2^ [σ^2^_1_ + σ^2^_2_])/∆^2^ [31] with alpha and power levels of 0.05 and 0.8, respectively. The mean difference (∆ = 0.43) and standard deviations (σ_1_ = 0.20, σ_2_ = 0.27) were calculated using data from a randomized controlled trial study [24]. As per calculation, the sample size per group was five cats, and this study was divided into eight groups. The total sample size was 40 cats. To prevent the missing completely at random, we added cats to any group by 50%, so eventually there were 80 total cats. A total of 80 healthy cats comprising 36 males and 44 females were enrolled and admitted for routine neutering. The ages ranged from 6 months to 6 years, with a median age of 1.4 ± 0.67 years. Most cats did not belong to any particular breed, with 81.25% comprising the domestic shorthair breed. Other breeds were Scottish Fold, Persian, Exotic, and Munchkin. Most of the cats had not been admitted to a veterinary clinic before, while only 28.75% had experienced time in a cage. A clinical examination by a veterinary surgeon including mucous membrane, capillary refill time, pulse and heart rate, respiratory rate, turgor test, body condition assessment, rectal temperature, abdominal palpation, and venipuncture to assess hematology (complete blood count) and serum biochemistry (total serum protein, creatinine, and alanine aminotransferase) indicated that all cats were healthy before enrollment. All cats were diagnosed as the American Society of Anesthesiologists Grade 1. All owners confirmed the general health of their cats a few days before the trial. The wards were physically separated from the dog area with a stainless-steel cage (90 cm width × 75 cm height × 90 cm length). The average temperature in the wards was 26.4°C. Each cage was furnished with a water and food bowl, a litter box, and bedding of soft pads. Three sides of the cage were covered with blankets, and there was no visible access to any other caged cat.

### Demeanor scoring system

A baseline value for cat demeanor was recorded immediately on admission. Before separation into the treatment groups, DSS was used to classify the cats. The examiners, which included veterinarians and students, underwent training for the DSS scoring system. The total DSS score was a simple sum of responses from all questions, with each question receiving a value ranging from 0 to 4 based on the selected option [29]. The cats were divided into two demeanor categories: DSS1 (friendly cats) and DSS2 (aggressive cats). The DSS1 group was comprised of friendly, confident, and shy cats, with scores ranging from 1 to 8, while the DSS2 group cats were withdrawn, protective, and aggressive, with scores ranging from 9 to 24. Friendly cats in the DSS1 group were loveable when handled. The withdrawn and aggressive cats in the DSS2 group remained ridged and appeared uneasy when handled. The DSS2 cats were ridged or frozen, while some demonstrated pawing, biting, or clawing when handled. Three examiners classified cat demeanor and were blinded to the treatment groups.

### Experimental protocol

After individually housing the cats in cages with no additional enrichment for 24 h, 39 cats were classified as DSS1 and 41 cats were classified as DSS2. On day 2, the surgery was performed. Open castration and ovariohysterectomy protocols were performed in male and female cats [32]. After surgery, cats from each group were randomly assigned to one of four treatment groups containing different environmental enrichment items, including the control or no additional enrichment in the cages (C), a hiding box (B), pheromone treatment (P), and a hiding box combined with pheromone treatment (BP). Treatment B (DSS1 n = 10, DSS2 n = 10) provided cats with a hiding box measuring 39 × 25 × 25 cm (L × W × H) at the back of the cage. In treatment P (DSS1 n = 10, DSS2 n = 10), the cat cages were placed in a 220 × 640 × 250 cm room, and in the internal area, a diffuser of synthetic analog of the feline facial pheromone fraction F3 (Feliway^®^, Ceva Santé Animale, Libourne, France) was installed at the height of 2.0 m. The pheromone diffuser was operated for 24 h for 7 days. In treatment BP (DSS1 n = 9, DSS2 n = 10), the cage contained a hiding box placed in the pheromone room, while for the control treatment (C) (DSS1 n = 10, DSS2 n = 11), the cage had no hiding box or pheromone diffuser. The caregivers were blinded to control bias from cointervention.

### Behavioral observation

Cat stress score, developed by Kessler and Turner [12], was used in this study to observe stress behaviors. This system evaluates the level of cat stress based on the posture of body elements such as the head, belly, tail, legs, eyes, pupils, whiskers, ears, and behavior such as activity and vocalization. The CSS scores ranged from 1 to 7 as follows: 1 = Fully relaxed, 2 = Weakly relaxed, 3 = Weakly tense, 4 = Very tense, 5 = Fearfully stiff, 6 = Very fearful, and 7 = Terrorized. The CSS scores were evaluated twice daily at 09.00 am and 05.00 pm on day 1 (before surgery) and on days 2–8 after surgery or admission. The first observer stood in front of the cages for 10 min, observing the cats, and another observer completed the assessment for each cat within 15 min. Both observers were trained to interpret cat body language and were familiar with the CSS criteria in a previous pilot study. The observers were blinded to control bias from outcome assessment.

The cages were separated into three sections (front, middle, and back) using adhesive tape as markers. The location of each cat within the cage was recorded by the examiners twice daily to determine the presence of hide-seeking behavior.

Food intake was recorded for all cats. Food and water remaining in the bowls from the previous day were removed and weighed before the daily feedings. The weight of each cat was recorded on day 1, day 3, and day 8.

### Saliva cortisol measurement

Immediately after behavioral observation on day 1 (24 h after admission), saliva was collected from the cats, with samples collected at the same time at 10 am on days 3 and 7 after admission. Three saliva samples were randomly assigned to each group in the C, B, P, and BP groups of DSS1 and DSS2. All candidate cats fasted from food and water for at least 2 h before collecting saliva. An absorbent ophthalmic sponge was introduced into the lateral commissure of the mouth or under the tongue and the cats were allowed to chew on the sponges until they were thoroughly moistened for a period of 1–2 min. The saliva sponges were immediately packed into sterile 1.5 mL microcentrifuge tubes containing an upside-down 10 μL pipette tip without touching to prevent contamination. The saliva samples within the small tubes were then centrifuged at 1000× *g* for 2 min and the saliva was collected for analysis. The samples were stored at −20°C until processing. An immunoassay (Cortisol EIA Kit, Boster Biological Technology, Pleasanton, CA, USA) was used to simultaneously measure cortisol levels in the samples. The sensitivity limit for the cortisol assays was 17.3 pg/mL, as provided by the manufacturer.

### Statistical analysis

Continuous variables were presented as mean ± standard deviation or median and interquartile range (IQR) after normal distribution testing by histogram and Shapiro–Wilk test. Categorical variables were presented as percentages, while the Kruskal–Wallis and Wilcoxon signed-rank tests were used to analyze the salivary cortisol levels between the groups. Multivariable linear regression was used to estimate the CSS, food intake, and body weight changes in all groups.

To estimate the position of the cats inside the cages for all groups, multinomial logistic regression was used. All statistical analyses were performed using Stata Statistical Software, version 15.0 (StataCorp LLC, College Station, TX, USA). Statistical significance was set at p < 0.05.

## Results

### Cat stress score in the DSS 1 group

**Figure-1:**
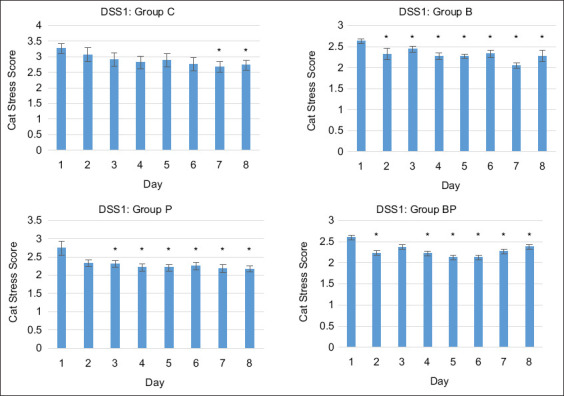
Relationship of median cat stress score with time for the friendly cat group (demeanor scoring system 1) and treatment with hiding box (B group), pheromone (P group), and hiding box together with pheromone (BP group). Significant differences between the 1^st^ and the other days are indicated by *p < 0.05.

During the observation days, the control group cats had higher CSS than the treatment groups. The median (IQR) in the control group was 2.76 (3.42–2.41), while for the B, P, and BP groups, the values were 2.28 (2.52–2.11), 2.27 (2.49–2.04), and 2.28 (2.47–2.10), respectively. Treatment cats had a significantly lower stress level than the control cats (p < 0.001). There was no significant difference in CSS among the treatment groups. The data had a non-normal distribution as verified by the Shapiro–Wilk test. Confounding variables were observed on the control day, and multivariable linear regression was used to compare CSS in the DSS1 group between the 1^st^ day and the 2^nd^ to the 8^th^ day. Cat stress score continuously decreased, with statistical significance on the 2^nd^, 3^rd^, and 2^nd^ day after admission in the B (p = 0.034), P (p = 0.017), and BP (p = 0.012) treatment groups, respectively. In the control group, a significant decrease in stress score was observed on the 7^th^ day of admission (p = 0.016) (Figure-1).

### Cat stress score in the DSS 2 group

**Figure-2:**
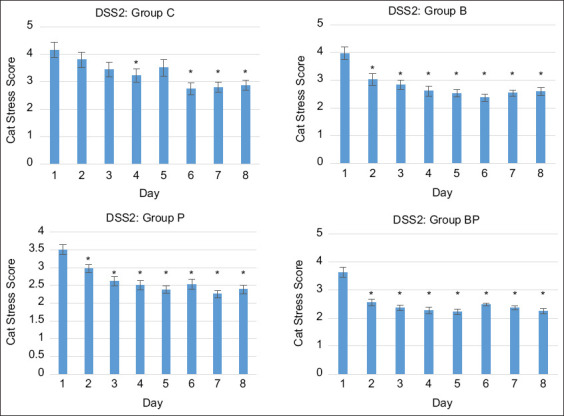
Relationship of median cat stress score with time for the aggressive cat group (demeanor scoring system 2) and treatment with hiding box (B group), pheromone (P group, and hiding box together with pheromone (BP group). Significant differences between the 1^st^ and the other days are indicated by *p < 0.05.

Significant differences were observed between the control and treatment groups in aggressive cats (DSS2). Especially on the 1^st^ day of admission, cats in the control group had the highest stress levels. Median (IQR) CSS values for the C, B, P, and BP groups on all the observation days were 3.21 (3.95–2.58), 2.7 (3.15–2.30), 2.49 (2.98–2.25), and 2.45 (2.68–2.22), respectively. When comparing the CSS between the 1^st^ day and the other days, CSS in the control group significantly decreased on day 6 (p < 0.001) of admission, whereas the B (p = 0.004), P (p = 0.005), and BP (p < 0.001) group scores significantly decreased on the 2^nd^ day. Significant differences in CSSs between the treatment groups were found between B (2.81) and P (2.64) (p = 0.043) and between B and BP (2.55) (p = 0.001). This result revealed that the pheromone reduced stress levels more than providing a hiding box. A hiding box and pheromone combination significantly lowered stress in aggressive cats (Figure-2).

### Position inside the cage

Differences in locations of the cats in the cages were observed between the 1^st^ day and the other days. Results showed that 40% of the P group cats and 60% of the B group cats in DSS1 spent more time in front of the cage than the cats in the control group (10%). Cats in the control group spent their time mostly at the back of the cage (50%) and in the litter box (40%). For DSS2 cats, there was no significant difference between the time cats spent in front of the cage in the P group cats compared to control cats, while cats in the P and PB groups spent their time in the front section of the cage more than the other groups. However, when comparing positions inside the cage between the control and treatment groups in both DSS1 and DSS2, multinomial logistic regression revealed no significant differences (p > 0.05).

### Food intake and body weight change

The data obtained had a non-normal distribution as verified by the Shapiro–Wilk test. In DSS1, the BP group recorded the highest food intake (median [IQR]; 67.5 [0–330]), while in DSS2, the control and BP groups had the most food intake (median [IQR]; C = 47.5 [0–135] and PB = 47.5 [0–140]). Quantile regression was used to compare food intake between the 1^st^ day and the 2^nd^ to the 8^th^ day. The DSS1 group showed no significant difference in food intake in the control group (p > 0.05). On the 3^rd^ day, food intake in the B and PB groups increased significantly, while in the P group, food intake increased significantly on the 7^th^ day. On the 3^rd^ day, in DSS2, the control and BP groups showed significant increases in food intake. On the 5^th^ day, food intake was significantly different in the B (p = 0.012) and P (p = 0.005) groups. Multivariable linear regression was used to compare body weight change between the control group and treatment group, with no significant differences (p > 0.05).

### Salivary cortisol

Among the admitted cats, the salivary cortisol levels varied. The median cortisol result was high on the 1^st^ day in the C and B groups, with a gradual decrease in saliva cortisol levels in the B group. A histogram and the Shapiro–Wilk test were used to test the data distributions, with results showing a non-normal distribution. Cortisol levels were analyzed between the groups at each time interval using the Kruskal–Wallis test. Results showed no differences between measured cortisol levels within each group during the same period. Using the Wilcoxon signed-rank test, data between each period were analyzed in each group. Results showed no differences between the intervals in each group, as shown in Table-1.

**Table-1:** Median (IQR) of salivary cortisol (ng/mL) in cats on days 1, 3, and 7 of admission for routine neutering surgery.

Observation day	Saliva cortisol level (ng/mL)			

	C (n = 3)	B (n = 3)	P (n = 3)	BP (n = 3)
1	1.21 (1.81–0.31)	1.83 (2.67–1.27)	0.83 (0.98–0.73)	0.65 (1.72–0.36)
3	1.01 (2.35–0.63)	1.48 (1.54–0.99)	1.59 (2.39–1.03)	1.26 (1.60–1.00)
7	1.23 (2.28–1.07)	1.15 (2.24–0.86)	1.53 (2.30–0.92)	1.38 (2.16–0.84)

C=Control group, B=Hiding box, P=Pheromone, BP=Hiding box and pheromone, IQR=Interquartile range

## Discussion

This study aimed to assess stress in hospitalized cats following neutering surgery, as well as the impact of providing environmental enrichment of a hiding box and/or the use of pheromone product to reduce stress. Numerous studies on the environment and welfare of domestic cats have been conducted on cats living in research facilities or shelters; however, a better understanding of stress in cats admitted to veterinary hospitals is needed. This study used a DSS to define the cat’s temperament before providing the treatments. Based on the knowledge that cats with diverse behavioral demeanors respond differently to different types of stressors or environmental enrichment, the cats were divided into two groups, DSS1 (friendly cat) and DSS2 (aggressive cat) [23]. According to the study’s results, the demeanor score affected cat stress levels, with DSS2 having a baseline significantly higher CSS than DSS1. In addition, cats with different demeanors responded to environmental enrichment differently. Environmental enrichment was more effective in aggressive cats than in friendly cats. We suggest including DSS as part of routine clinical practice to improve cat management during hospital visits with medical- or surgical-related processes.

Cat stress score created by Kessler and Turner [12] is commonly used to monitor stress over time, whether as a reaction to a new environment or to compare stress levels among the treatment and control groups. Cat stress score has been used to monitor stress in cats in a variety of studies over the last few years, including changes in shelter and sociability [33], relationships between owner and cat behavior [34], evaluation of environmental enrichment to reduce stress in cats admitted to veterinary clinics [35], and technology for new perspectives in cat welfare [36]. The mean CSSs of the DSS1 and DSS2 groups between 2.27 and 3.21 corroborate with the findings of Kessler and Turner [12] and Paz *et al*. [35]. Cat stress scores were highest on the 1^st^ day of admission. Traveling to the veterinary clinic and being introduced to a novel environment are stressful experiences for cats [6]. In our study, providing a hiding box for DSS1 and DSS2 cats reduced CSS, with aggressive cats showing a faster reduction in stress scores than friendly cats. An intrinsic part of the biology of a cat is the hiding behavior. The results were similar to a previous study that found that cats provided with a hiding box were able to rapidly reduce CSS in cats with a new shelter environment [37].

The pheromones are mainly received by the vomeronasal sensory neurons in the vomeronasal organ (VNO). The VNO transduces pheromonal signals to the amygdala and hypothalamus, inducing either a physiological or behavioral response [38]. A feline facial pheromone analog diffuser was previously advised for use in the consulting room to alleviate stress in cats visiting a veterinary clinic [39]; however, no research has assessed the use of pheromone in cats after surgery. As veterinary clinics may receive stimuli from other pets that can induce stress in cats, all cats in our study were kept in a dedicated room. This study has differing results from Doonan *et al*. [40], who described the effects of Feliway^®^ on feline stress during veterinary examination. They found no statistical significance between the use of Feliway^®^ and placebo wipes; however, they suggested that it may be more useful in combination with environmental enrichment and proper handling techniques by the examiner [40]. Silva *et al*. [16] found that saliva cortisol levels decreased in 75% of cats after exposure; however, no difference was observed between before and after pheromone exposure. They hypothesized that animals were susceptible to the pheromone in different ways or spent varying amounts of time in areas with pheromone devices. In friendly cats, Feliway^®^ statistically reduced stress scores on day 3, while a combination of Feliway^®^ and a hiding box reduced stress scores on the 2^nd^ day. The aggressive cat group showed reduced stress scores on the 2^nd^ day of treatment with pheromone, with or without a hiding box. When comparing stress levels in aggressive cat groups between hiding box alone and hiding box with pheromone, the combination was found to be more effective in reducing stress. Therefore, results supported the use of Feliway^®^ to reduce cat stress, with varying effects on cats with different temperaments.

Playing a crucial role in the control of intermediate metabolism and stress, cortisol is a hormone/steroid that has long been used to assess HPA. In serum, cortisol is found in free and protein-bound forms, but only in the free form in saliva [41]. Cortisol level has been used as a biomarker to monitor stress in animals, such as in cats after the administration of gabapentin [17], wildlife to assess animal welfare [42], piglets after weaning [43], and dogs at home or in veterinary clinics [44]. In this investigation, saliva sampling was chosen as the method to measure cortisol concentration because it is non-invasive and requires less restraint for cats than venipuncture [18]. The salivary collection approach also effectively reflects the biologically active component of cortisol. Cortisol levels in saliva can be accurately determined because bound cortisol does not readily diffuse in saliva and concentrations are relatively unaffected by saliva flow rate [45]. However, since the circulating half-life of cortisol is quite short and varies between 70 and 120 min [46], the timing of saliva sampling in this study may result in no significant differences in cortisol levels. Samples were collected without grouping cats of various demeanors. This research included surgery and propofol anesthesia for cats. The previous study has shown that drugs do not influence cortisol levels in saliva [18]. Individual cats also have diverse physiological responses to stress, such as physical characteristics (coat length, coat color, size, breed, and demeanor), previous experience, age, sex, and environments [47]. Cortisol levels varied significantly between individuals and at various sample times throughout the day [18]. Saliva cortisol samples were collected at the same time daily. However, for the small sample size, no differences in cortisol levels were found between each experiment group. The inclusion of only three samples per treatment group of DSS1 and DSS2 cats was a major constraint in this investigation, which may have impacted the discovery of significant differences between the groups. The lack of a difference in cortisol concentration in this study does not help to differentiate stress levels in hospitalized cats clearly. A more effective method to evaluate stress and animal emotions is infrared thermography (IRT). The IRT, a technique for measuring electromagnetic radiation emitted by bodies, was used to assess early inflammatory processes, neoplasms, and autonomic nervous system responses associated with stress-induced hyperthermia [44–50]. This method, together with cortisol level evaluation and behavioral observation, provides a more accurate stress analysis in hospitalized cats.

## Conclusion

This is the first study to combine a demeanor score system with stress scores in cats following surgery and hospitalization. The various effects on friendly and aggressive cats when provided with a simple environmental enrichment in the cat cage were discussed. Using both DSS and CSS to evaluate stress in hospitalized cats, the results show that the use of efficient techniques and providing hiding boxes and/or pheromones reduce stress and improve the welfare of cats. These enrichments induced a rapid stress reduction, especially in aggressive cats. Environmental enrichment helps cats adapt to their surroundings while also improving animal welfare.

## Data Availability

The supplementary data can be available from the corresponding author on a reasonable request.

## Authors’ Contributions

WK, TJ, and MS: Conceptualized and designed the study and drafted and revised the manuscript. WK, RM, SR, KR, and SW: Carried out the experiment. All authors have read and approved the final manuscript.
